# The intensification of metallic layered phenomena above thunderstorms through the modulation of atmospheric tides

**DOI:** 10.1038/s41598-019-54450-1

**Published:** 2019-11-29

**Authors:** Bingkun Yu, Xianghui Xue, Chengling Kuo, Gaopeng Lu, Christopher J. Scott, Jianfei Wu, Ju Ma, Xiankang Dou, Qi Gao, Baiqi Ning, Lianhuan Hu, Guojun Wang, Mingjiao Jia, Chao Yu, Xiushu Qie

**Affiliations:** 1 CAS Key Laboratory of Geospace Environment, Department of Geophysics and Planetary Sciences, University of Science and Technology of China, Hefei, China; 2 Department of Meteorology, University of Reading, Berkshire, UK; 3 Mengcheng National Geophysical Observatory, School of Earth and Space Sciences, University of Science and Technology of China, Hefei, China; 4 CAS Center for Excellence in Comparative Planetology, Hefei, China; 5 Institute of Space Science, National Central University, Jhongli, Taiwan; 6 State Key Laboratory of Numerical Modeling for Atmospheric Sciences and Geophysical Fluid Dynamics (LASG), Institute of Atmospheric Physics, Chinese Academy of Sciences, Beijing, China; 7 Key Laboratory of Earth and Planetary Physics, Institute of Geology and Geophysics, Chinese Academy of Sciences, Beijing, China; 8 State Key Laboratory of Space Weather, Center for Space Science and Applied Research, Chinese Academy of Sciences, Beijing, China; 9 Key Laboratory of Middle Atmosphere and Global Environment Observation, Institute of Atmospheric Physics, Chinese Academy of Sciences, Beijing, China; 10 Collaborative Innovation Center on Forecast and Evaluation of Meteorological Disasters, Nanjing University of Information Science and Technology, Nanjing, China

**Keywords:** Atmospheric chemistry, Space physics

## Abstract

We present a multi-instrument experiment to study the effects of tropospheric thunderstorms on the mesopause region and the lower ionosphere. Sodium (Na) lidar and ionospheric observations by two digital ionospheric sounders are used to study the variation in the neutral metal atoms and metallic ions above thunderstorms. An enhanced ionospheric sporadic *E* layer with a downward tidal phase is observed followed by a subsequent intensification of neutral Na number density with an increase of 600 cm^−3^ in the mesosphere. In addition, the Na neutral chemistry and ion-molecule chemistry are considered in a Na chemistry model to simulate the dynamical and chemical coupling processes in the mesosphere and ionosphere above thunderstorms. The enhanced Na layer in the simulation obtained by using the ionospheric observation as input is in agreement with the Na lidar observation. We find that the intensification of metallic layered phenomena above thunderstorms is associated with the atmospheric tides, as a result of the troposphere-mesosphere-ionosphere coupling.

## Introduction

Ionospheric sporadic *E* (*E*_*s*_) and neutral sodium (Na) layers are both metallic layered phenomena of great interest in the Mesosphere/Lower Thermosphere (MLT) region. The *E*_*s*_ layers are thin and dense patches of metallic ions at altitudes between 90 and 130 km. The metallic ions result from meteoric ablation^1–4^. The metallic *E*_*s*_ formation relies on the vertical wind shear provided by the tidal wind, i.e., mostly the diurnal and semidiurnal tides^5–8^. The *E*_*s*_ descends with the vertical downward tidal phase until it weakens and then becomes depleted below 100 km. The neutralization of Na^+^ occurs through three-body reactions needed for the metallic ions to form cluster ions, followed by increased dissociative electron recombination. The lifetime of Na^+^ rapidly decreases from several days above 100 km to only a few minutes at 90 km^4^. Therefore, the mesospheric sodium exists as layers of neutral atoms at altitudes of 80–105 km with a peak number density of 10^3^–10^4^ cm^−3^ near 92 km. There are also high temporal and spatial correlations between the *E*_*s*_ and sporadic Na layer^9,10^.

The coupling of tropospheric thunderstorms with the upper atmosphere and ionosphere has been known for many years since the pioneering work of *Wilson*^11^. A thunderstorm can disturb the MLT region through convective atmospheric gravity waves (GWs)^12,13^ and lightning-induced transient electromagnetic phenomena^14–23^. The speculative connection between thunderstorms and the *E*_*s*_ layer was proposed in the 1930s^24,25^ and was first reported in Nature magazine based on a statistical superposed epoch analysis (SEA) from a very large dataset during the period from 1993 to 2003^15^. It was the first paper to reveal an enhancement of the *E*_*s*_ layer which occurs ~6 h and ~30 h after lightning. After that, more studies applied the same methodology of SEA on lightning and *E*_*s*_ layer measurements using hourly lightning events as trigger times^18,26–29^. At present, the mechanism responsible for the ionospheric *E*_*s*_ perturbations above thunderstorms is not well understood. A serious difficulty in explaining the lightning-induced coupling phenomena is the observed large lag time between the lightning trigger events and the most statistically significant *E*_*s*_ response. The lag time ranges from several hours to more than 30 hours^15,26,29^. Recent work reported a novel observation of the meteoric metals in the mesosphere, and atomic Na layer, that is significantly intensified ~19 h after lightning^30^. Nearly all these studies of lightning-induced effects on the metallic layered phenomena were statistical results obtained using the SEA method, and it is difficult to explain such time lags by GWs or electrodynamic effects induced by lightning alone. Recently, two case studies of mesoscale convective storms that moved through two ionosonde stations were reported^31^, in which a reduction and then disappearance of the ongoing *E*_*s*_ layer was observed.

Gravity waves are discounted as a plausible mechanism on the basis that the large time delay between lightning and Es layers of 6–more than 30 hours far exceeded the propagation time expected for GWs travelling up from the troposphere^32^. As mentioned above, the enhancement of Na layer is closely related to the occurrence of *E*_*s*_ layer. In our study, a multi-instrument experiment combined with the observation of neutral metal atoms and metallic ions was carried out in order to explore lightning-*E*_*s*_-Na relations. It was found that the lightning-induced intensification of metallic layered phenomena is associated with atmospheric tides, which modulate the troposphere-mesosphere-ionosphere coupling. The large time delay in previous statistical studies could be explained by the tidal periodicities in the *E*_*s*_ variability. Furthermore, Na cluster ion chemistry is also considered in to be part of the process involved in the intensification of metallic layered phenomena. A Na chemistry model using the ionospheric *E*_*s*_ observation as input was used to compare the Na number density in simulation with Na lidar observations. The intensification of metallic layered phenomena above thunderstorms is modulated by diurnal and semidiurnal tides. We further discuss the dynamical and chemical processes in the *E*_*s*_ layer and Na layer during thunderstorms, suggesting the possible role of thunderstorms in this coupling of the neutral atmosphere and ionosphere.

## Results

### Observations of metal atoms and ions

The World-Wide Lightning Location Network (WWLLN) used in this study is a global lightning detection system^33^. It has a relatively high detection efficiency in east Asia. At present, it is one of the best suited global lightning detection systems for investigating the location of the intense lightning (>50 kA) and its influence on the upper atmosphere.

The Chinese Meridian Project is a ground-based multi-station chain along 120°E longitude to monitor the atmosphere and space environment^34^. Observations of the neutral metal Na atom layers used in this study are measured by a broadband dye-laser-based Na resonance fluorescence lidar at Haikou, China (20.0°N, 110.3°E), as part of the Chinese Meridian Project. The lidar has been routinely operated since 2010. A total of 1,577 h Na layer observations on 197 nights from 2010 to 2013 are available.

Ionospheric data were obtained using two digisondes near the Haikou Na lidar station. These were Digital Portable Sounder 4D (DPS-4D) digisondes at Sanya (18.3°N, 109.6°E) and at Fuke (19.4°N, 109.0°E). These ionospheric observations can provide simultaneous information of metallic ions during the routine Na lidar operation.

**Figure 1:**
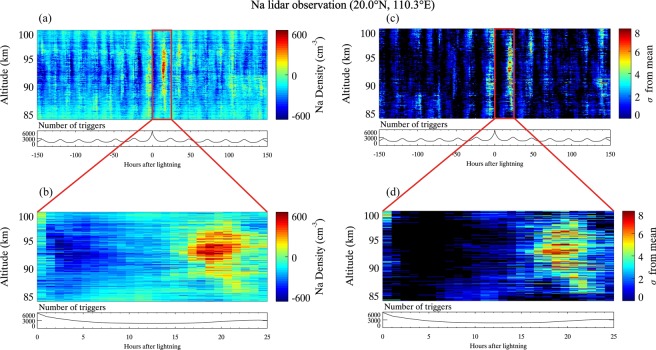
The enhancement of the neutral metal Na layer in response to lightning, observed by the Na lidar at Haikou (20.0°N, 110.3°E). (**a**) The residual of Na median number density from superposed epoch analyses 150 h before and after lightning trigger time, after removing the diurnal trend. (**b**) The zoom-in window in (**a**) between 0 and 25 h. (**c**) the significance of the resulting change in Na number density in response to lightning. The colour scale represents the number of standard deviations from the mean before lightning. (**d**) The zoom-in window in (**c**) between 0 and 25 h.

It has been previously revealed that there is an enhancement of the neutral Na layer in response to lightning^30^. The data from these 28 thunderstorm nights are further analyzed here, to investigate the possible mechanism, based on the Na lidar data and ionospheric data from two digisondes. Figure 1a shows the median in neutral metal Na number density over a range of altitudes for 150 hours either side of the lightning trigger events, after removing the diurnal trend to exhibit the response of Na layer to lightning. In this SEA, the lightning trigger times occur at time = 0. The number of triggers occuring at each time is plotted in the panel below. It can be seen that, while all lightning events occur at time = 0, the recurrent nature of thunderstorms means that there is a diurnal variation in lightning events evident in the occurrence of lightning throughout the SEA. This is echoed in the residual diurnal variation seen in the variation of Na number density before and after time = 0. The Na layer number density is significantly intensified up to 600 cm^−3^ after lightning. The zoom-in window between 0 and 25 h plotted in red is shown in Fig. 1b. The enhancement of Na layer is evident at 17–24 h after lightning between 85 and 98 km, with the maximum increase of ~600 cm^−3^ at t = 19 h, at an altitude of 93 km.

Figure 1c,d show the significance of the resulting change in Na number densities. It can been seen that there is a distinct region between t = 0–25 h in which the confidence value is >5*σ*. There are some other small patches of 3–4*σ* level because of the recurrent nature of thunderstorms and the relatively low signal-to-noise ratio at higher and lower heights resulting from the observational limits for lidar.

**Figure 2:**
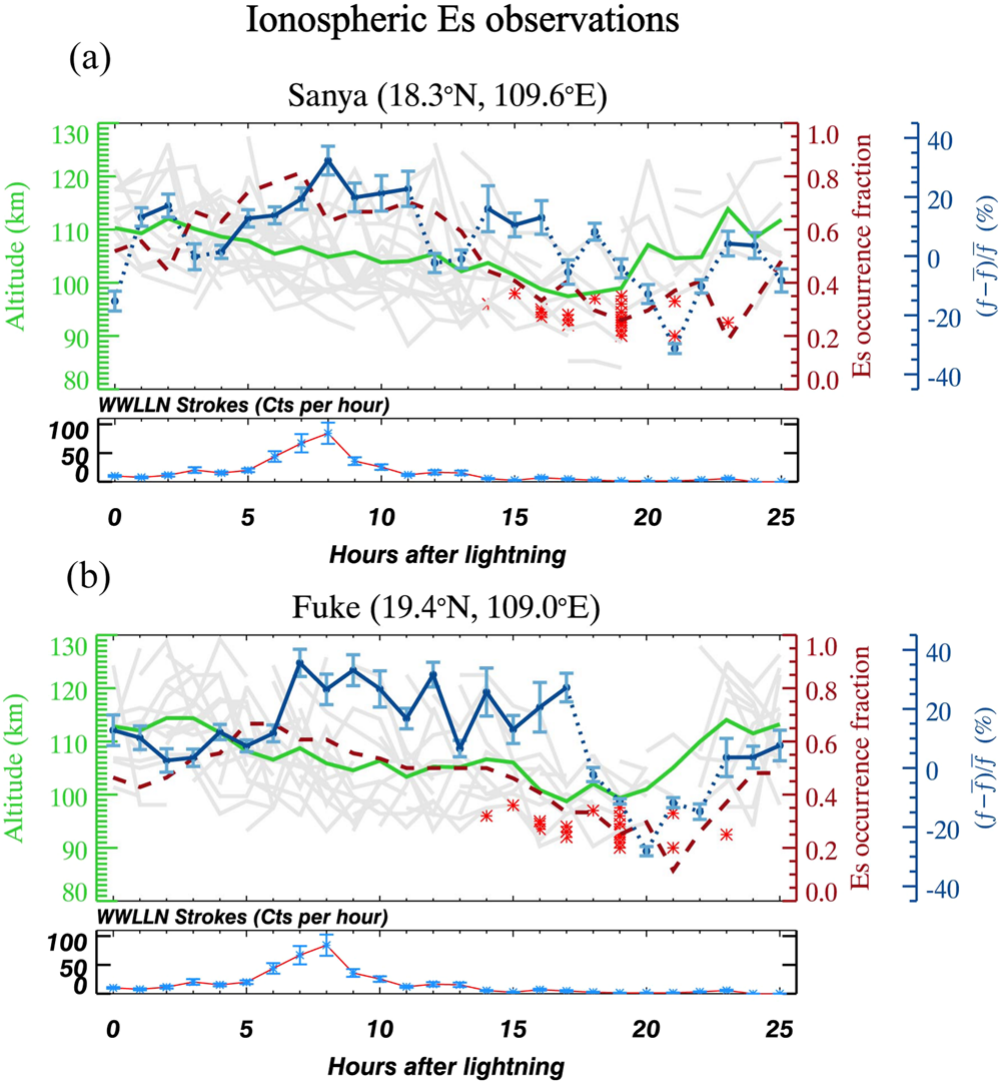
The ionospheric observations by two digisondes at Sanya (18.3°N, 109.6°E) and Fuke (19.4°N, 109.0°E). The change in *δh*′*Es* each night is plotted as grey lines. The mean *δh*′*Es* value is shown as a green line. The mean occurrence fraction of *E*_*s*_ is shown as a red dashed line. Mean relative (negative and positive) changes of *f*_0_*E*_*s*_ ($(f-\bar{f})/\bar{f}$) are shown as blue (dotted and solid) lines, with the standard error in these mean values. Red stars show the altitude and time of maximum Na concentration on each thunderstorm night, measured by a Na lidar at Haikou (20.0°N, 110.3°E). The lower panels are the average rate of WWLLN strokes, as an indicator of the intensity of thunderstorms.

However, the hourly cadence of the lightning data used in this study is insufficient to record the development of thunderstorms. In this study, we consider the average rate of WWLLN strokes as an indicator of the intensity of thunderstorms, as shown in Fig. 2. It has previously been shown that there are high correlations between the *E*_*s*_ and sporadic Na layer^9^. Figure 2a shows all the time series of hourly ionospheric *E*_*s*_ layer observations on 28 thunderstorm nights at Sanya. The time ranges from 0 to 25 hours after trigger time. The lower panel is the average number of WWLLN strokes per hour as an indicator of the intensity of thunderstorms. The average duration of thunderstorms is ~13 h (from 10.11 Cts/hour at t = 0 h to 15.43 Cts/hour at t = 13 h), much longer than usual continental thunderstorms. There are more intense lightning strokes over the coastal Haikou lidar station on the Hainan island, with the 2,534.15 J mean stroke energy, 1,197.20 J median stroke energy, 31.3% high energy strokes (>2,000 J) and 13.2% low energy strokes (<400 J), compared with the continental area, e.g., Beijing with the 2,128.47 J mean stroke energy, 632.38 J median stroke energy, 19.3% high energy and 34.4% low energy strokes for the same period. Although on average the enhancement of Na number density occurs at t = 19 h, the observed time that each Na layer reaches its maximum differs each night. The altitude and time of maximum Na concentration over the 28 nights are marked by red stars. All the time series of hourly profiles of *E*_*s*_ height are plotted in grey lines. The average occurrence fraction of *E*_*s*_ during the 28 nights is plotted in red dashed lines. The *E*_*s*_ layer occurs more frequently during thunderstorms. The relative change of critical frequency, *f*_0_*E*_*s*_ ($(f-\bar{f})/\bar{f}$) is shown in blue dotted and solid lines, in which $\bar{f}$ is the average background frequency of *f*_0_*E*_*s*_. Since *f*_0_*E*_*s*_ is a function of layer height^35^, for each point $\bar{f}$ was calculated from all layers within our dataset which had a similar height (within ± 0.5 km). These were then subtracted from each time after lightning in our study. Blue solid lines are used when two consecutive points are positive, and blue dotted lines are used otherwise. The error bar of the relative change of frequency is calculated from the standard error over the 28 nights of data.

The *E*_*s*_ layer is statistically enhanced as shown in Fig. 2a, which is in agreement with previous studies^15^. Both the occurrence fraction and relative change of *E*_*s*_ seem to vary with the development of the underlying thunderstorms, when using the average rate of WWLLN strokes as an indicator of the intensity of thunderstorms. The variation in *E*_*s*_ is positively related to the average rate of WWLLN strokes. Between 0–8 h there is an increase in the occurrence of lightning strokes, during which the *E*_*s*_ layer becomes more dense and frequent. The peak of the observed relative *f*_0_*E*_*s*_ reaches ~35% (the average *f*_0_*E*_*s*_ at the peak is 5.03 MHz) and the maximum of the *f*_0_*E*_*s*_ occurrence fraction is ~0.80. Between t = 8 and 13 h, both the critical frequency and occurrence fraction of the *E*_*s*_ layer decrease, along with the decrease in thunderstorm activity. The *f*_0_*E*_*s*_ reaches its peak 8 hours after the beginning of the thunderstorms (t = 0 in the SEA), which is close to the time delay of 6 hours between the thunderstorm activity and the response of the *E*_*s*_ ^15^. The time delay of 6 hours may be a result of differences in definition of the thunderstorm activity (initial lightning occurrence or peak lightning stroke rate in the SEA). It has been suggested that the gravity waves generated during thunderstorms and nonlinear GW breaking effects can contribute several hours to the *E*_*s*_ temporal variability^32^. The height of *E*_*s*_ layer decreases until it descends below 100 km before the Na number density reaches the maximum value. The *E*_*s*_ height descent is mainly driven by tidal winds which provide the vertical wind shear to shepherd the ions downwards through their tidal phase velocity propagation. To clearly show the tendency, the average height of *E*_*s*_ is plotted in a green solid line. The height of *E*_*s*_ shows a diurnal variation, mainly controlled by the diurnal tide.

Figure 2b shows data from another digisonde at Fuke near the Na lidar. The *f*_0_*E*_*s*_ increases with the thunderstorm activity. The peak of the relative *f*_0_*E*_*s*_ reaches 30–35% (the average *f*_0_*E*_*s*_ at the peak is 5.02 MHz) and the maximum of the *f*_0_*E*_*s*_ occurrence fraction is ~0.67. The occurrence fraction of *E*_*s*_ measured at Fuke reaches its maximum before the peak of the thunderstorm activity. It could be likely a result of the competitive effects on the occurrence fraction of *E*_*s*_, with a development of thunderstorms and a decrease in height of *E*_*s*_. It shows the similar tendency of tide-period height. With the decrease in the *E*_*s*_ height below 100 km and the occurrence of the maximum neutral Na layer during thunderstorms, a remarkable reduction of relative *f*_0_*E*_*s*_ and then disappearance of the *E*_*s*_ with a decrease in occurrence fraction are observed as reported^31^. Furthermore, Fig. 2b shows the *E*_*s*_ height is controlled not only by the diurnal tide but also by the semidiurnal tide.

### Chemical simulation

In addition to the dynamics in the *E*_*s*_ and Na layers which are mostly controlled by the diurnal and semidiurnal tides, the role of chemistry in the MLT region should also be considered in the decay of a descending *E*_*s*_ layer and the related occurrence of an intensified Na layer. To study the Na reactions with the *E*_*s*_ layer, a Na chemistry model is used to simulate the dynamical and the chemical processes of the enhanced Na layer through the modulation of atmospheric tides. The neutral and ionic gas-phase chemistry schemes in our Na model are taken from the reactions of Na neutral chemistry and ion-molecule chemistry with their rate coefficients in the Table 1, based on a recent atmospheric chemistry review^4^.

**Table 1:** Reactions of Sodium Neutral Chemistry and Ion-molecule Chemistry with their Rate Coefficients in the Model^4^.

	Reaction	Rate coefficient
**Neutral Chemistry**		
R1	$Na+{O}_{3}\to NaO+{O}_{2}$	$1.1\times {10}^{-9}exp(\,-\,116/T)$
R2	$NaO+O\to Na+{O}_{2}$	$(2.2\times {10}^{-10}){(T/200)}^{1/2}$
R3	$NaO+{O}_{3}\to Na+2{O}_{2}$	$3.2\times {10}^{-10}exp(\,-\,550/T)$
R4	$NaO+{H}_{2}\to NaOH+H$	$1.1\times {10}^{-9}exp(\,-\,1100/T)$
R5	$NaO+{H}_{2}\to Na+{H}_{2}O$	$1.1\times {10}^{-9}exp(\,-\,1400/T)$
R6	$NaO+{H}_{2}O\to NaOH+OH$	$4.4\times {10}^{-10}exp(\,-\,507/T)$
R7	$NaOH+H\to Na+{H}_{2}O$	$4\times {10}^{-11}exp(\,-\,550/T)$
R8	$NaOH+C{O}_{2}\,(\,+\,M)\to NaHC{O}_{3}$	$(1.9\times {10}^{-28}){(T/200)}^{-1}$
R9	$NaHC{O}_{3}+H\to Na+{H}_{2}C{O}_{3}$	$(1.84\times {10}^{-13}){T}^{0.777}exp(\,-\,1041/T)$
R10	$Na+{O}_{2}\,(\,+\,M)\to Na{O}_{2}$	$(5.0\times {10}^{-30}){(T/200)}^{-1.22}$
R11	$Na{O}_{2}+O\to NaO+{O}_{2}$	$5.0\times {10}^{-10}exp(\,-\,940/T)$
R12	$2NaHC{O}_{3}\,(\,+\,M)\to {(NaHC{O}_{3})}_{2}$	$(8.8\times {10}^{-10}){(T/200)}^{-0.23}$
**Ion-Molecule Chemistry**		
R20	$Na+{{O}_{2}}^{+}\to N{a}^{+}+{O}_{2}$	$2.7\times {10}^{-9}$
R21	$Na+N{O}^{+}\to N{a}^{+}+NO$	$8.0\times {10}^{-10}$
R22	$N{a}^{+}+{N}_{2}\,(\,+\,M)\to Na\cdot {{N}_{2}}^{+}$	$(4.8\times {10}^{-30}){(T/200)}^{-2.2}$
R23	$N{a}^{+}+C{O}_{2}\,(\,+\,M)\to Na\cdot C{{O}_{2}}^{+}$	$(3.7\times {10}^{-29}){(T/200)}^{-2.9}$
R24	$Na\cdot {{N}_{2}}^{+}+X\to Na\cdot {X}^{+}+{N}_{2}\,(X=C{O}_{2},{H}_{2}O)$	$6\times {10}^{-10}$
R25	$Na\cdot {{N}_{2}}^{+}+O\to Na\cdot {O}^{+}+{N}_{2}$	$4\times {10}^{-10}$
R26	$Na{O}^{+}+O\to N{a}^{+}+{O}_{2}$	$1\times {10}^{-11}$
R27	$Na\cdot {O}^{+}+{N}_{2}\to Na\cdot {{N}_{2}}^{+}+O$	$1\times {10}^{-12}$
R28	$Na\cdot {O}^{+}+{O}_{2}\to N{a}^{+}+{O}_{3}$	$5\times {10}^{-12}$
R29	$Na\cdot {Y}^{+}+{e}^{-}\to Na+Y(Y={N}_{2},C{O}_{2},{H}_{2}O,O)$	$(1\times {10}^{-6}){(T/200)}^{-1/2}$
R30	$N{a}^{+}+{e}^{-}\to Na+h\nu$	$(3.9\times {10}^{-12}){(T/200)}^{-0.74}$

The model runs over 30 days for all species in order to reach a steady state, and then the ionospheric observations from the digisonde at Fuke, near the Na lidar station, from t = 0 to 25 h are input to drive the Na model. The *E*_*s*_ layer is assumed to be a Gaussian height profile (*σ* = 3 km) with maximum electron concentration calculated from *f*_0_*E*_*s*_. The electron concentration of the *E*_*s*_ layer in cm^−3^ is estimated from *f*_0_*E*_*s*_ in MHz, by the formula *N*_*e*_ = 1.24 × 10^4^ × *f*^2^. The ionospheric observations are input with the time interval of 5 minutes, using linear interpolation between adjacent hourly observed values. Rocket-borne mass spectrometer measurements have proved that the *E*_*s*_ layer is mostly the ionization of metal atoms, in which ~4% of ions measured are the Na^+^ ions^36,37^. The variation in height of the *E*_*s*_ layer is also considered as the dynamical process of plasma in the Na chemistry model. As the ion species in the simulations are driven by the ionosonde observation, here we simplify the vertical transport of neutral species as $\frac{\partial ({N}_{X}w)}{\partial z}$, in which w represents the vertical wind velocity and *N*_*X*_ represents the major neutral gasphase sodium species, Na and NaHCO_3_. The term is added in the left of Eqs. (2) & (3) as the vertical transport of neutral species^38,39^.The vertical wind for diurnal and semidiurnal tides is retrieved from the Global-Scale Wave Model 2009 (GSWM-09) tidal climatologies^40,41^.

**Figure 3:**
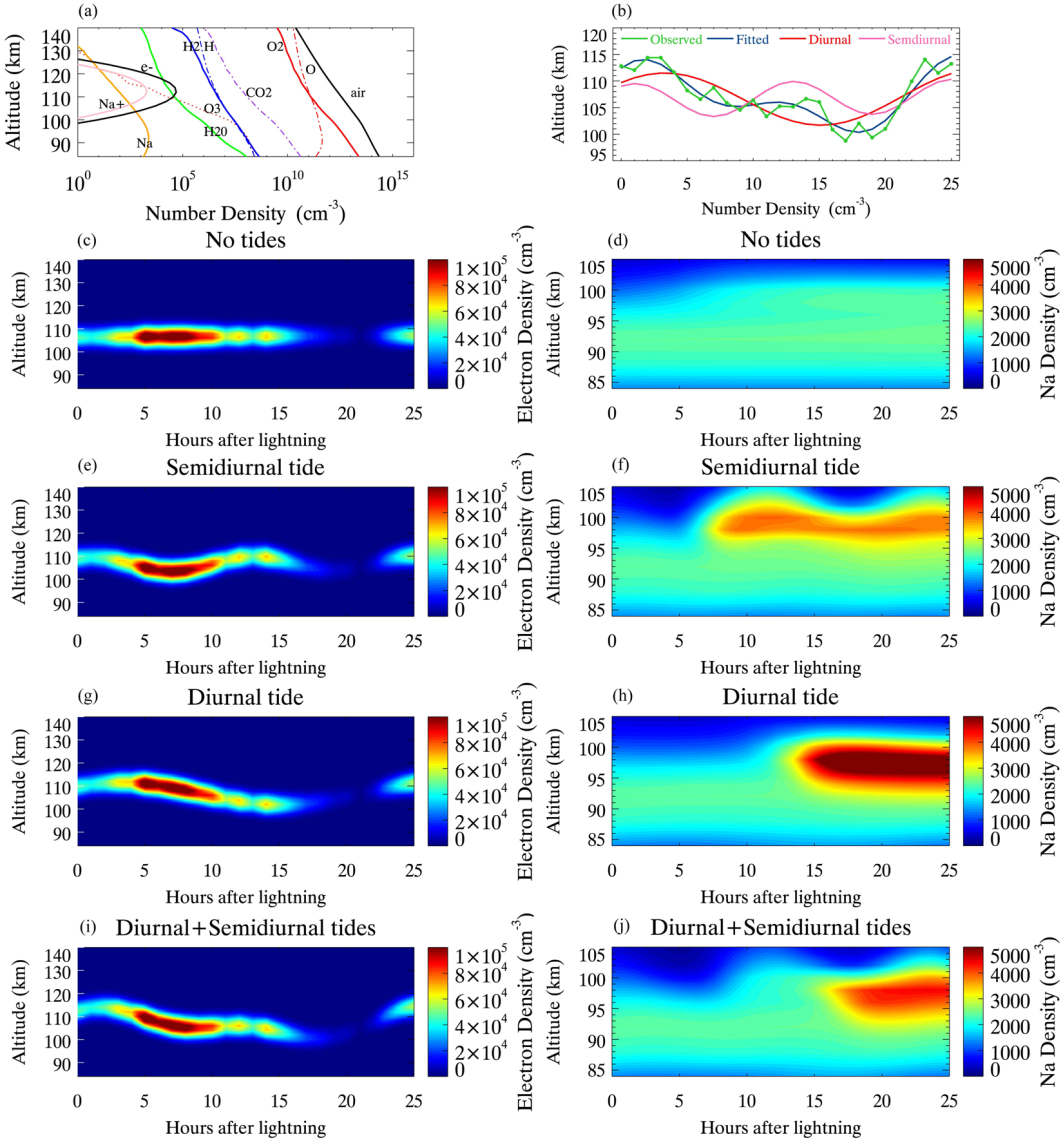
Na chemical simulations (**a**) the background of concentrations of chemical species and simulated number density profile of Na at t = 0 h. (**b**) The green and blue lines correspond to the observed variation in *E*_*s*_ height and its harmonic fit, consisting of corresponding diurnal (red lines) and semidiurnal (pink lines) components. The electron density of *E*_*s*_ layer and the simulated Na number density are shown with no tides (**c,d**), semidiurnal tide (**e,f**), diurnal tide (**g,h**) and diurnal + semidiurnal tides (**i,j**), respectively.

The background concentrations of chemical species and simulated density profiles of Na and Na^+^ at t = 0 h are shown in Fig. 3a. The average variation in height of *E*_*s*_ during 28 nights observed by the Fuke digisonde is plotted as green lines in Fig. 3b. The blue lines represent the harmonic fit consisting of corresponding diurnal (red lines) and semidiurnal (pink lines) components. Figure 3c–j are the simulation results from the Na model, in which there were (1) no tides, (2) semidiurnal tide, (3) diurnal tide, and (4) diurnal + semidiurnal tides.

Figure 3c,d show the electron concentration of the *E*_*s*_ layer and the simulated Na number density without modulation of tides. The *E*_*s*_ layer maintains an altitude of ~106 km without tidal perturbations. There is a weak response in the Na layer because the *E*_*s*_ does not descend with the tidal phase. The number density of the background Na layer is approximately 2,400 cm^−3^, which is consistent with the average Na lidar observations at Haikou.

In the case of Fig. 3e,f, the *E*_*s*_ layer is modulated by a semidiurnal tide. The height of *E*_*s*_ shows a semidiurnal tidal periodicity in Fig. 3e. The *f*_0_*E*_*s*_ increases with the thunderstorms and the height of the *E*_*s*_ layer descends below 105 km. When a reduction of *E*_*s*_ occurs, the Na layer is obviously enhanced because of the high ion-neutral collision frequencies at low altitudes leading to an enhanced loss of Na^+^ ions. The maximum Na number density is 3,800 cm^−3^ at t = 8 h, at an altitude of 98 km and 3,800 cm^−3^ at t = 20 h, at an altitude of 98 km.

Figure 3g,h show the electron concentration of the *E*_*s*_ layer and the simulated Na number density with diurnal tide. The height of *E*_*s*_ shows a diurnal variation. The enhancement of Na layer occurs at t = 19 h and at an altitude of 98 km, with maximum value of 5,800 cm^−3^.

Note that the cases with a semidiurnal or diurnal tidal component alone cannot reflect the actual variation in the height of *E*_*s*_ observed by the digisonde. The downward phase propagation of the node could be ahead of or behind the observed phase. Actually, the *E*_*s*_ layer descends with the vertical downward tidal phase, mostly by both diurnal and semidiurnal tides^5–8^. Figure 3i,j show the exact case with diurnal and semidiurnal tides. The height of *E*_*s*_ is controlled by the diurnal and semidiurnal tides. The *E*_*s*_ layer descends with the vertical tidal phase until it weakens and is depleted below 100 km at t = 18 h. The simulated Na layer is significantly enhanced with its value of ~4,200 cm^−3^ at t = 19 h, and at an altitude of 98 km.

From our results we conclude that atmospheric tides play dominant roles in the dynamical process of the *E*_*s*_ and Na layer. Diurnal and semidiurnal perturbations in the structure or abundance of Na layer could be found. Note that the Na chemistry model is driven by the ionospheric *E*_*s*_ observations and the monthly GSWM-09 tidal climatologies only. No tuning parameters were used in the simulation. Monthly averaged diurnal and semidiurnal tides from the GSWM are considered in the Na chemistry model with other tidal components and GWs sources are neglected. In the downward motion of the lightning-induced enhanced *E*_*s*_ layer, following the tidal vertical phase, the Na layer intensifies as metallic Na^+^ ions and electrons recombine. The Na^+^ ions are neutralized through three-body reactions, followed by a subsequent enhancement of dissociative electron recombination.

The fitted height of *E*_*s*_ in Fig. 3i is in good agreement with the observed average variation in height of *E*_*s*_ observations over 28 nights. Figure 3j shows the simulated response of the Na layer during thunderstorms in which the Na layer is modulated by diurnal and semidiurnal tides. The model reproduces an enhancement of the Na layer number density at 19 h, which is consistent with the Na lidar observation. The simulation results show a strong relationship between a descending *E*_*s*_ layer and the occurrence of an ehanced Na layer. This simulation provides a possible mechanism for the intensification of Na layer ~19 h after lightning^30^. The lightning-induced enhanced *E*_*s*_ layer descends with the tidal phase below 100 km. Then the *E*_*s*_ depletes via enhanced three-body collisional recombination to form the enhanced metal Na layer above thunderstorms. An enhanced Na layer during thunderstorms seems to be associated with an intensification of the *E*_*s*_ layer being driven by the modulation of atmospheric tides. The modulation of atmospheric tides dominates the variation in the *E*_*s*_ layer.

We then include both the horizontal and vertical transport $\nabla \cdot ({N}_{X}\overrightarrow{v}(u,v,w))$ in the left of Eqs. (2) & (3) considering that the magnitude of horizontal gradient of mass flux $({N}_{X}\overrightarrow{v}t)$ is comparable with the vertical gradient of mass flux^39^:

1 $$\nabla \cdot ({N}_{X}\overrightarrow{v})=\frac{\partial ({N}_{X}u)}{\partial x}+\frac{\partial ({N}_{X}v)}{\partial y}+\frac{\partial ({N}_{X}w)}{\partial z}$$

where u, v and w represent the zonal, meridional and vertical wind velocities. The horizontal density gradients of Na and NaHCO_3_ in this simulation are adopted by the output from Whole Atmosphere Community Climate Model (WACCM) with a metal chemistry module for Na^42,43^. WACCM is global chemistry-climate model framed by the Community Earth System Model^44^ developed by the National Center for Atmospheric Research.

**Figure 4:**
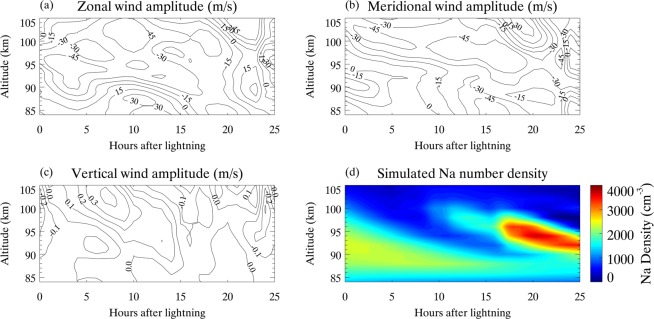
Variations in (**a**) zonal wind, (**b**) meridional wind, (**c**) vertical wind from SD-WACCM, and (**d**) the simulated Na number density.

The effects of background winds on the *E*_*s*_ layers and neutral Na layers include the horizontal and vertical transport of the involved either ion and neutral sodium species, as well as the *E*_*s*_ height descent induced by the downward progression of semidiurnal and diurnal tides. As mentioned above, based on the combination of ionospheric *E*_*s*_ measurements from the ground-based ionosonde and climatological GSWM model, we are able to produce a lightning-induced intensification of Na layer with 19 h delay after lightning. For this simulation case study, a specific dynamics (SD) version of WACCM is used, in which winds and temperaure fields below 50–60 km are nudged towards the Goddard Earth Observing System Model, Version 5 (GEOS-5)^45^. Figure 4a–c shows the variations in three-dimensional wind field in the simulation using output from SD-WACCM^46^ with 144 vertical model levels, increasing the resolution to ~500 m in the MLT. As we stated earlier, the metallic ions densities observed by the digisonde shown in Fig. 3b are input to drive the Na model. In addition to the neutral vertical transport of major gasphase sodium species, the horizontal transport induced by zonal and meridional winds is included. Figure 4d shows the simulated Na number density. The enhancement of Na layer occurs at t = 19 h and at an altitude of 94 km, with maximum value of 3,400 cm^−3^. The maximum increase of Na density is ~1,000 cm^−3^ with a downward tidal phase, which is consistent with the Na lidar observations.

As mentioned above, the statistical results of various studies using the SEA method have demonstrated that thunderstorm activity can affect *E*_*s*_ layers^15,18,26–29,31^. The lightning-induced response of *E*_*s*_ layers is found to occur several hours after lightning, which makes it difficult to explain these phenomena solely by thunderstorm-generated GWs or lightning-emitted electromagnetic pulses. In fact, the very large time delay in previous statistical studies could be explained by the tidal periodicities in the *E*_*s*_ variability. A 24-h diurnal tidal modulation could explain the very large lag times observed between lightning and the response of the *E*_*s*_ layer in previous statistical studies. For example, *E*_*s*_ intensifications were observed to occur ~6 h and ~30 h after lightning^15^. In our study, both the occurrence fraction of and peak density of *E*_*s*_ seem to vary with the development of the underlying thunderstorms, when we consider the average rate of WWLLN strokes as an indicator of the intensity of thunderstorms. It is found the similar lag time in that the peak in the relative *f*_0_*E*_*s*_ occurs ~8 h after the lightning trigger time, comparable to ~6 h^15^. The Na model reproduces an enhancement of Na layer through diurnal and semidiurnal tides. We conclude that the atmospheric tide is a dominant dynamical process in the MLT and plays dominant roles in the formation and intensification of metallic layered phenomena above thunderstorms.

## Discussion

**Figure 5:**
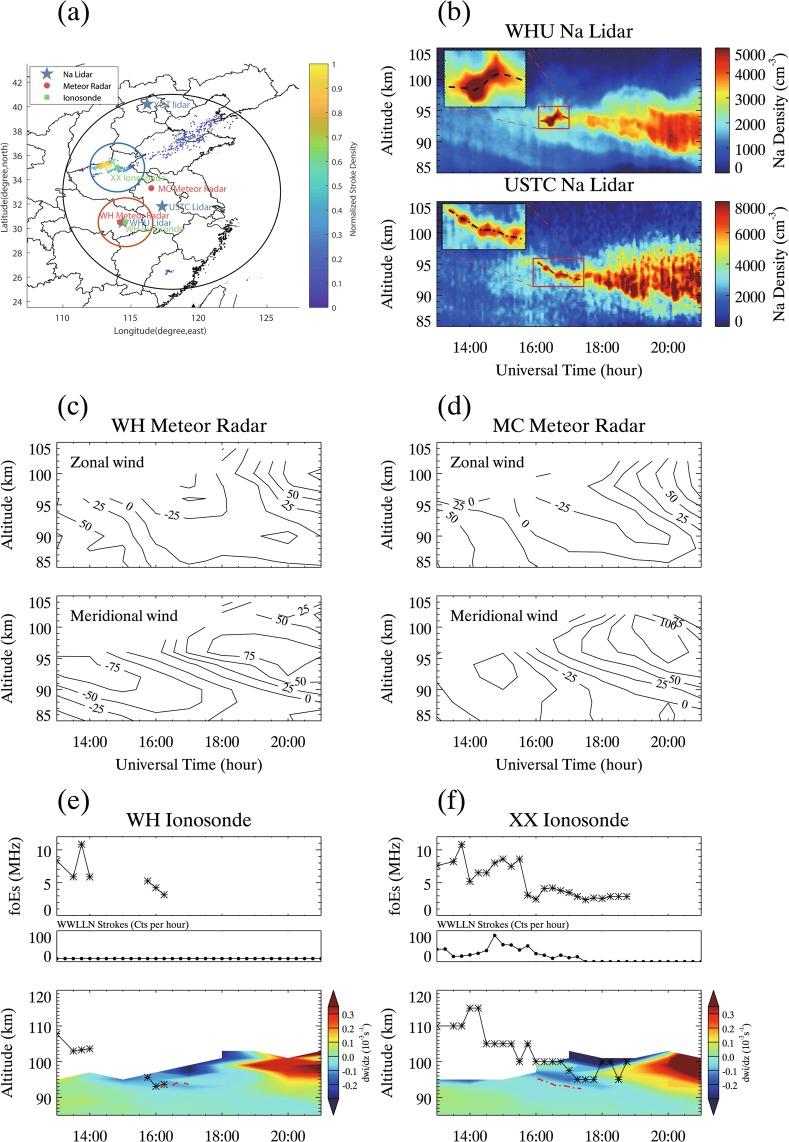
The multi-instrument observations of sporadic E layers and sporadic Na layers at mid-latitudes on 3rd August 2015. (**a**) the experimental instruments at mid-latitudes under the Chinese Meridian Project. The blue stars represent three Na lidar locations (the University of Science and Technology (USTC) Na lidar (31.8°N, 117.3°E), Wuhan University (WHU) Na lidar (30.5°N, 114.4°E), and Yanqing (YQT) Na lidar (40.2°N, 116.2°E)). The red circles represent meteor two all-sky meteor radars locations (the Wuhan (WH) meteor radar (30.5°N, 114.2°E) and Mengchen (MC) meteor radar (33.3°N, 116.5°E)). The green crosses represent two ionosondes locations (the Wuhan (WH) ionosonde (30.5°N, 114.6°E) and Xinxiang (XX) ionosonde (35.0°N, 114.0°E)). The colored points show the normalized stroke density during thunderstorm and lightning activities. (**b**) Na number density co-observed by the WHU and USTC Na lidars. The zoom-in window plotted in red shows the sporadic Na layer cases with the centroid heights plotted in dashed or dashed-dotted lines. (**c**,**d**) the zonal and meridional wind observed by the WH and MC meteor radars. (**e**,**f**) the simultaneous observations of *E*_*s*_ layers by the WH ionosonde and XX ionosonde. The upper panels show the critical frequency *f*_0_*E*_*s*_ and WWLLN stroke rate during thunderstorms. The bottom panels denote the downward motion of *E*_*s*_ layers and the following related centroid heights of Na layers, which is consistent with downward moving convergent node of tidal winds (negative values in the calculated divergence of vertical ion velocity dw_*i*_/dz). The red dashed and dashed-dotted lines represent the centroid heights of sporadic Na layers.

The sporadic E layers and sporadic Na layers both have a small vertical scale of several kilometers and a large horizontal scale of hundreds to thousands of kilometers^47,48^. We analyzed the multi-instrument ground-based obeservations of an enhanced *E*_*s*_ layer, followed by a subsequent enhanced Na layer above a thunderstorm on 3rd August 2015^49^. Figure 5a shows the mid-latitude experimental instruments under the Chinese Meridian Project. The ground-based facilities are three Na lidars (the University of Science and Technology (USTC) Na lidar (31.8°N, 117.3°E), Wuhan University (WHU) Na lidar (30.5°N, 114.4°E), and Yanqing (YQT) Na lidar (40.2°N, 116.2°E)), two all-sky meteor radars (the Wuhan (WH) meteor radar (30.5°N, 114.2°E) and Mengchen (MC) meteor radar (33.3°N, 116.5°E)), and two ionosondes (the Wuhan (WH) ionosonde (30.5°N, 114.6°E) and Xinxiang (XX) ionosonde (35.0°N, 114.0°E)). The colored points show the normalized stroke density during thunderstorm and lightning activities. A severe thunderstorm is observed over XX ionosonde. In Fig. 5b, the Na number densities co-observed by the USTC and WHU Na lidars exhibit a similar morphology, with an occurrence of a sporadic Na layer between 16:00–17:30 shown in the zoom-in window. The YQT Na lidar did not operate during that night. This sporadic Na layer event induces the following increase in the Na densities of the main Na layer between 17:30–21:00, which, in the USTC lidar, is higher than the Na densities in the WHU lidar. The horizontal distance between the USTC lidar and WHU lidar is ~310 km, which indicates the Na layer has a large horizontal scale. The centroid heights of the sporadic Na layer are plotted in dashed and dashed-dotted lines, respectively. Figure 5c,d show the wind fields observed by the WH meteor radar and MC meteor radar, which is used to investigate the relationship between the occurrence of the enhancement of Na layers with downward motion of metallic ions and the downward moving convergent nodes of tidal winds. Figure 5e,f represent the simultaneous observations of *E*_*s*_ layers by the WH ionosonde and XX ionosonde. The upper panels show the critical frequency *f*_0_*E*_*s*_ and WWLLN stroke rate during thunderstorms. The *E*_*s*_ layer existed over a long period of time and remained at a relatively large *f*_0_*E*_*s*_ in Xinxiang, compared with the *E*_*s*_ layer in Wuhan. There are intense lightning strokes over Xinxiang and the maximum stroke rate is 85 Cts/hour. The bottom panels in Fig. 5e,f show the divergence of vertical ion velocity and the variations in the heights of *E*_*s*_ and the centroid heights of sporadic Na layers. The occurrence of sporadic Na layers is located within a downward moving convergent node of ions, which is associated with a *E*_*s*_ layer descent. This result confirms the role of downward moving convergent nodes of atmospheric tidal winds in the descending lightning-induced enhanced *E*_*s*_ layer and subsequent enhancement of Na layers.

Thunderstorms influence the upper atmosphere and the ionosphere both enhancing ionisation^14,15,18,29,50^ and causing ionospheric depletions^31^. The influence of thunderstorms on the ionospheric *E*_*s*_ layer around 100 km was first identified using the SEA method^15^. It is proposed that the variation in metal atoms may be related to lightning-associated intensification of *E*_*s*_ layer above thunderstorms. It remains possible that the observed lightning-induced enhancement in the *E*_*s*_ is associated with TLEs^26^. The magnitude of the perturbation in the *E*_*s*_ layer is also associated with the intensity of lightning^29^. The electrical processes related to lightning and sprites above thunderstorms may have roles in the coupling of the atmosphere and ionosphere^51^.

**Figure 6:**
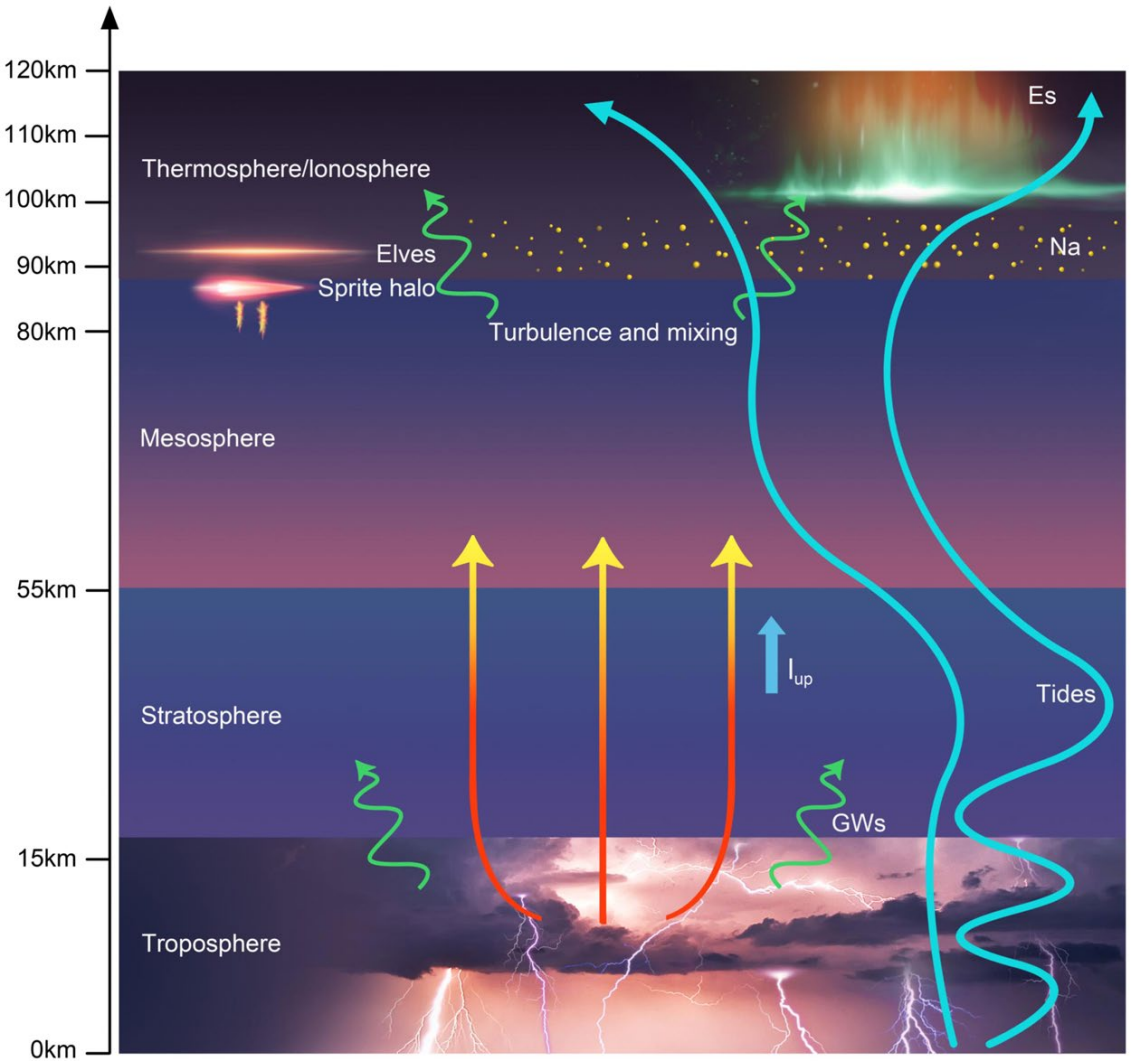
Schematic diagram illustrating the proposed mechanism for the lightning-associated enhancement of *E*_*s*_ and neutral Na layer during thunderstorms mainly modulated by atmospheric tides, and potentially influenced by the thunderstorm electrical effects and gravity waves (GWs) induced by the tropospheric thunderstorms.

Tropospheric thunderstorms can also affect the upper atmosphere through GWs induced in the lower atmosphere^12^. Atmospheric tides modulate the dynamical process in the MLT and play important roles in the dynamical process of the metallic layered phenomena, such as the *E*_*s*_ layer^5,6,8^ and the neutral metal Na layer^9,52–54^. In principle, GWs could also alter and influence the tidal forcing^8^. Tidal backgrounds in the mesosphere could be reinforced through nonlinear interactions with GWs^55,56^. The luminosity of elves with pronounced stripes was also found to be modulated by the thunderstorm-induced GWs^13^. Therefore, the thunderstorm-generated GWs would be expected to act to influence the coupling processes of atmosphere and ionosphere. In Fig. 6, a schematic diagram is shown to illustrate the proposed mechanism for the thunderstorm influence on the ionospheric *E*_*s*_ layer and neutral metal Na layer.

In this study, we present a combination of observational and numerical modelling results. The results presented indicate that the thunderstorm electrical effects could accelerate and enhance the process in which metallic Na^+^ ions are lost to form neutral Na atoms. We conclude that the increase in the concentration of neutral Na atoms 19 h after lightning could be attributed to the enhanced ionospheric *E*_*s*_ layer during thunderstorms. Atmospheric tides control the dynamical process in the MLT region and the *E*_*s*_ layer descends with a diurnal tidal downward phase. The descending lightning-induced enhanced *E*_*s*_ layer becomes depleted below 100 km. In this downward motion, the three-body collisions become more effective and the chemical lifetime of Na^+^ decreases. It efficiently enhances the dissociative electron recombination, to form the enhanced neutral Na layer above thunderstorms.

However, the whole coupling processes of atmosphere and ionosphere above thunderstorms has not been comprehensively proven, and it is left as an open question as by which mechanism thunderstorms influence the upper atmosphere. More investigations are needed to further study these questions using both observations and modelling.

## Methods

### Na chemistry model

In the Na model, the *E*_*s*_ layer is initialized with a selected percentage of Na^+^ and then descends at the rate observed by the digisonde. The redistribution and rates of neutralization of Na^+^ and e^−^, and changes in the main long-lived Na species, such as Na, NaHCO_3_ are determined from the solution of continuity equations (summarised in Eqs. 1–4), while other short-lived intermediates are considered as a steady-state concentration^57^. During the *E*_*s*_ descent, the fresh Na atoms are produced through three-body reactions in which the metallic ions form cluster ions which undergo dissociative electron recombination. Wave action and vertical eddy diffusion are included in the model. The time tendencies of Na, NaHCO_3_, Na^+^ and e^−^ are described as follows:

2 $$\frac{d[Na]}{dt}={I}_{abl}+A[NaHC{O}_{3}]+B[N{a}^{+}]-(C+D)[Na]-\nabla {\Phi }^{Na}$$

3 $$\frac{d[NaHC{O}_{3}]}{dt}=D[Na]-A[NaHC{O}_{3}]-2{k}_{12}{[NaHC{O}_{3}]}^{2}-\nabla {\Phi }^{NaHC{O}_{3}}$$

4 $$\frac{d[N{a}^{+}]}{dt}=C[Na]-B[N{a}^{+}]-\nabla {\Phi }^{N{a}^{+}}$$

5 $$\frac{d[{e}^{-}]}{dt}=-\,B[N{a}^{+}],$$

where *I*_*abl*_ is the Na injection rate determined from the height profiles of the Na input rate from meteor ablation, calculated for the Long Duration Exposure Facility meteoroid size distribution^58^ (85% reduction in all mass ranges with a global input of 12.1 tons day^−1^) with the peak at an altitude of 94 km^57^.

$$\begin{array}{rcl}A & = & {k}_{9}[H],\\ B & = & {k}_{22}[{N}_{2}][M](\frac{{k}_{24}([C{O}_{2}]+[{H}_{2}O])+{k}_{29}[{e}^{-}]+\frac{{k}_{29}[{e}^{-}]{k}_{25}[O]}{{k}_{26}[O]+{k}_{27}[{N}_{2}]+{k}_{28}[{O}_{2}]+{k}_{29}[{e}^{-}]}}{{k}_{24}([C{O}_{2}]+[{H}_{2}O])+{k}_{29}[{e}^{-}]+{k}_{25}[O]\frac{{k}_{26}[O]+{k}_{28}[{O}_{2}]+{k}_{29}[{e}^{-}]}{{k}_{26}[O]+{k}_{27}[{N}_{2}]+{k}_{28}[{O}_{2}]+{k}_{29}[{e}^{-}]}})\\  &  & +\,{k}_{23}[C{O}_{2}][M]+{k}_{30}[{e}^{-}],\\ C & = & {k}_{20}[{O}_{2}^{+}]+{k}_{21}[N{O}^{+}],\\ D & = & ({k}_{1}[{O}_{3}]+{k}_{10}[{O}_{2}][M])(\frac{{k}_{4}[{H}_{2}]+{k}_{6}[{H}_{2}O]}{{k}_{2}[O]+{k}_{3}[{O}_{3}]+{k}_{4}[{H}_{2}]+{k}_{5}[{H}_{2}]+{k}_{6}[{H}_{2}O]})(\frac{{k}_{8}[C{O}_{2}][M]}{{k}_{7}[H]+{k}_{8}[C{O}_{2}][M]}).\end{array}$$

We neglect the day-time photochemical reactions, to study the process of an enhanced Na layer associated with the *E*_*s*_ layer on thunderstorm nights. The Na model is one-dimensional, from 50–140 km with an altitude resolution of 2 km. Equations (1)–(4) are integrated with a 1 min time step. ∇Φ^*X*^ is the divergence of vertical flux of species X^57^. Φ^*X*^ is a function of height z:

6 $${\Phi }^{X}=-\,{K}_{zz}(\frac{\partial [X]}{\partial z}+[X](\frac{1}{H}+\frac{1}{T}\frac{\partial T}{\partial z})),$$

where *K*_*zz*_ is the vertical eddy diffusion coefficient, 4 × 10^5^ cm^2^ s^−1^. For the general nighttime condition over Haikou in July 2012, the background concentrations of chemical species and temperature are obtained from the WACCM with the metal chemistry included^59^.

## Data Availability

The data that support the findings of this study are available from the corresponding author upon reasonable request.
